# Use of peripheral electrical stimulation on healthy individual and patients after stroke and its effects on the somatosensory evoked potentials. A systematic review

**DOI:** 10.3389/fneur.2022.1036891

**Published:** 2022-11-18

**Authors:** Marko Mijic, Andres Jung, Benedikt Schoser, Peter Young

**Affiliations:** ^1^Department of Neurology, Friedrich-Baur-Institute, Klinikum der Universität, Ludwig-Maximilians-University, Munich, Germany; ^2^Institute of Health Sciences, Universität zu Lübeck, Luebeck, Germany; ^3^Clinic for Neurology, Medical Park, Bad Feilnbach, Germany

**Keywords:** peripheral electrical stimulation, somatosensory evoked potentials (SEP), stroke rehabilitation, sensory and motor recovery, somatosensory cortex

## Abstract

**Introduction:**

To date, a few studies have used somatosensory evoked potentials (SEP) to demonstrate cortical sensory changes among healthy subjects or to estimate cortical plasticity and rehabilitation prognosis in stroke patients after peripheral electrical stimulation (PES) intervention. The primary aim was to systematically review whether PES has a role in changing latencies and amplitudes of SEPs in healthy subjects and stroke patients. Moreover, we searched for a correlation between sensory and motor function assessments and changes in SEP components of included studies.

**Methods:**

The following databases were searched: Pubmed/MEDLINE, Scopus/ScienceDirect, Web of Science/Clarivate, Cochrane Library, The Physiotherapy Evidence Database (PEDro), and ClinicalTrials.gov. Titles and abstracts, as well as full-text reports, were screened for eligibility by two independent reviewers according to a priori defined eligibility criteria. There were no study limitations concerning the treatment of the upper limb, lower limb, or torso with PES.

**Results:**

The final systematic search resulted in 11,344 records, however only 10 were evaluated. We could not find enough evidence to confirm use of SEP as a predictor to estimate the rehabilitation prognosis after stroke. However, we found a correlation between different sensory and motor function assessments and changes in SEP components. The stroke studies involving PES that initiate a voluntary contraction used for a specific movement or task indicate a positive relationship and correlation to assessments of motor function. It could be indicated that PES have a predictive impact of sensory reorganization, as mirrored by the change in SEP amplitude and latency. However, it is not possible to verify the degree of connectivity between SEP and cortical plasticity. To confirm this hypothesis, we propose the conduction of randomized controlled trials in healthy volunteers and stroke patients.

**Systematic review registration:**

https://doi.org/10.17605/OSF.IO/U7PSY.

## Introduction

Peripheral electric stimulation (PES) is a rehabilitative technology that uses electrical currents to the peripheral nerves. It has been proposed that somatosensory stimulation in the form of electromyographically triggered neuromuscular electrical stimulation to the peripheral nerve can influence functional measures of motor performance in stroke patients and can additionally produce changes in cortical excitability (1, 2). In this way, PES provides restoration of walking or arm movements in individuals with complete or incomplete spinal cord injury, stroke, or other upper motor neuron lesion (2–4).

The literature offers multiple terms for peripheral electrical stimulation: transcutaneous electric nerve stimulation (TENS) (5–8), functional electrical stimulation (9–12), cutaneous electrical stimulation (13), somatosensory stimulation (14), neuromuscular electrical stimulation (1, 15) or combination of terms “percutaneous” and “neuromodulation”.

Sheffler and Chae (16) devoted important consideration to the use of electrical stimulation for motor relearning. They described three types of electrical stimulation available for motor learning: functional electrical stimulation (FES), electromyography or biofeedback mediated FES, and application of neuroprostheses. In the first case the patient is a passive participant in the FES training and no cognitive investment is necessary. The second type of exercises combines afferent feedback information with FES induced repetitive movements. During training with neuroprosthesis, functional tasks can be performed (2). The multiple PES terms used in the present review can be classified into one or more stimulation types described by Sheffler and Chae. Furthermore, for all terms the same technique is being used: placing surface electrodes on the skin overlaying sensory-motor nerve structures, establishing an electric field between two electrodes and ions, generating a current in the tissue. In the following text, only the term PES will be used exclusively.

In many studies, it has been found that the stroke patient's walking speed, endurance, and coordination improved with the use of PES (2, 17–19). The same modality on motor cortical excitability is described by recording motor evoked potentials (20–22), transcranial magnetic stimulation (23, 24) or fMRI (1, 25). On the other hand, the influence of PES on somatosensory function has been frequently overlooked in clinical context and research in the field of stroke rehabilitation (15). Prediction of upper limb (UL) and lower limb (LL) motor recovery in stroke patients is generally based on clinical examination (26). The prognosis is typically based on clinical impression, incorporating clinical and demographic factors such as stroke severity and age (27). Moreover, clinicians cannot know whether the prognoses they make at the acute stage are correct unless they do not routinely assess each of their patients several months later (27). This gestalt approach can produce differing opinions about prognosis and these seem to produce variation in discharge planning (28). According to Feys et al. (26) the combination of the motor score and somatosensory evoked potentials (SEPs) is best able to predict an outcome especially in the acute stroke phase, since neurophysiological measures alone are of limited value in predicting a long-term effect. The finding by Kato et al. (29) who examined the SEPs of the median and the tibial nerves in patients with hemorrhagic lesions, confirmed that 60 out of 65 arms (92.3%) and 50 out of 62 legs (80.6%) showed abnormalities in SEPs. These findings may indicate SEP measures quantifying latencies, thresholds, and evoked responses at high stimulator intensities had high reliability and require small sample sizes to power a study adequately (30). Therefore, the validation of SEP as a new standard neurophysiological tool for assessing the rehabilitation prognosis after stroke seems a reasonable decision.

SEPs are time-locked potentials evoked by electric stimulation of the sensory or mixed peripheral nerves and recorded along with the large fiber somatosensory (dorsal column–medial lemniscus) pathway. SEPs record transmission of an electrical signal/action potential between recording sites along the impulse pathway, thereby allowing the identification of abnormalities that help to localize a lesion (31).

Urasaki et al. (8) found that the dorsal column nucleus has the main role in CNS sensory amplification and that PES suppresses this amplification phenomenon in the medial lemniscus pathway. Consequently, use of PES as an intervention to verify SEP as a new standard neurophysiological tool could be a good method to observe changes in cortical somatosensory pathways.

The effects of PES on somatosensory cortical representation in healthy subjects have not been fully investigated yet, since little is known about the functional features of mismatch deviant and standard responses across different sensory brain modalities (32). This controversy of sensory changes and cortical plasticity persists in stroke patients and changes in corticomotor excitability still remains elusive (33).

To our knowledge, the role of PES in changing latencies and amplitudes of SEPs in healthy subjects or whether effects of PES aiming at motor rehabilitation after stroke have an impact on the improvement of pathological SEPs have not yet been studied. Furthermore, one of the aims of the present study was to examine the evidence on sensorimotor assessment and changes in SEP components after PES treatment for clinical correlations, so that SEP can be used at best as a predictor for estimating rehabilitation prognosis after stroke.

## Materials and methods

The study protocol was prospectively registered at the open science framework (OSF) with the registration DOI: https://doi.org/10.17605/OSF.IO/YW6PT on the 14th of March 2021 and an update protocol was registered in OSF (https://doi.org/10.17605/OSF.IO/U7PSY) on the 27th of August 2022. The PICO (34) model was implemented to answer the primary clinical questions: Do the effects of PES on motor rehabilitation in post-stroke patients have an impact on the latencies and amplitudes of pathological SEPs and does PES alter the latencies and amplitudes of SEPs in healthy subjects? (Table 1).

**Table 1:** PICO criteria.

P	Patient/Subjects	Healthy subjects Stroke patients
I	Intervention	Transcutaneous electric nerve stimulation, functional electrical stimulation, cutaneous electrical stimulation, somatosensory stimulation, neuromuscular electrical stimulation or combination of terms “percutaneous” and “neuromodulation”
C	Comparison	No PES intervention, placebo, inactive intervention, or waiting-list
O	Outcome	Latency and amplitude of somatosensory evoked potentials

This systematic review was conducted using “The Preferred Reporting Items for Systematic reviews and Meta-Analyses (PRISMA) statement 2020” (35) and followed recommendations from the Cochrane handbook (36).

### Study selection

A team of four healthcare professionals, including two physiotherapists (MM, AJ), and two physicians (PY, BS), established the study's aim, its primary outcome measures, the search strategy, and its eligibility criteria. The search construct consisted of two main subjects: peripheral electric stimulation and somatosensory evoked potentials. The following databases were searched: Pubmed/MEDLINE, Scopus/ScienceDirect, Web of Science/Clarivate, Cochrane library database, The Physiotherapy Evidence Database (PEDro), and ClinicalTrials.gov. The cut-off date of the search was the 28th of August 2022.

Because only 8 eligible studies were identified with the initial search, it was decided to repeat the search, include additional databases, use a revised search strategy, and publish an update registration protocol (see above). All search strategies (from the first search and from the update search) can be found in each registration protocol in OSF. Screening of all articles published in English and German was performed independently by two authors (MM, AJ) using the Rayyan QCRI software (37) and no automation tools were used in the process. The research team defined inclusion and exclusion criteria in advance. Reference sections of relevant review and research articles were used to identify additional pertinent articles. The full text of articles identified by the title and/or abstract as possibly applicable was retrieved, and the final decision on the inclusion was made by both reviewers independently. Disagreements between reviewers were resolved by consulting a third and fourth reviewers (PY, BS). The mesh term SEP was introduced in 1982 by PubMed, consequently the search time limit was set from January of the same year. If required, additional information was requested from the article authors. Data regarding the number of probands, study design, duration of treatments, and PES adjustments (Table 2) were extracted from each report. In addition, the researchers evaluated all found cortical latencies and amplitudes of SEPs fragments. The data of SEP fragments from healthy individuals and from patients with stroke regarding PES are presented separately to avoid misunderstanding of the evaluated fragments (Tables 3, 4).

**Table 2:** Peripheral electrical stimulation effect—characteristic summary.

**Study**	**Study design**	**Healthy or stroke population; no. of participants**	**Duration of treatments**	**Location of peripheral electrical stimulation**	**Form of stimulation; pulse amplitude; pulse duration; pulse frequency**	**Outcome measures**
**Studies made on healthy volunteers**						
Ashton et al. (6)	Case-matched study; three group; pre-post test	32 healthy volunteers; Group A/Placebo (11n); Group B/TENS (10n); Group C/Aspirin (11n)	TENS 5 cycles randomly varied between 30 and 33 stimuli ~5 min duration	TENS with two 8 cm^2^—disposable electrodes were placed on the ventral surface of the forearm between the elbow and the wrist.	Monophasic electric shock stimulation; Not Provided (individual); 0.2 ms; 100 Hz	Not provided
Cogiamanian et al. (38)	One-group; pre-post test	12 healthy volunteers; Group A/(12n) tsDCS + (5n) (Placebo) same volunteers as in group A	Transcutaneous spinal (anodal and cathodal) direct current stimulation for 15 min	2 pair of saline-soaked synthetic sponge electrodes placed on tenth thoracic spinal vertebra and other above the right shoulder.	Constant current pulses; 2.5 mA; Not provided; Not provided	Not provided
Schabrun et al. (33)	(Crossover model) One-group; pre post test	13 healthy volunteers; Motor Movement PES Intervention and Sensory PES 100 Hz Interventions	Each subject participated in two sessions (30 min of PES) separated by at least 72 h	On each occasion, a different electrical stimulation intervention was administered to the right ABP	Constant current pulses; **1**. Motor movement: Stimulus intensity set to sufficient to induce a mid-range thumb abduction; 0.1 ms; 30 Hz; **2**. Sensory 100 Hz: set at the point where the subject first reported perception of the stimulus; 0.1 ms; 100 Hz	TMS, MEP, EEG
Kang et al. (39)	(Crossover model) one-group; pre-post test	20 healthy volunteers; Sham TENS 2 Hz; TENS 2 Hz EA	The application of sham TENS, 2 Hz TENS and 2 Hz EA lasted for 15 min	Sham TENS and TENS electrodes were placed on the fibular side of the tibial tuberosity (electrode size is not provided)	Bidirectional symmetric square-wave pulses; 12 to 24 mA; Not provided; 2 Hz	Not provided
Rocchi et al. (40)	One-group; pre-post test	15 healthy volunteers	Subjects underwent 45 min of HF-RSS	Stimulation was delivered separately to the third phalanx of the right and left thumb and index finger using surface electrodes separated by 0.5 cm (anode placed distally to the cathode)	Constant current stimulator in the form of square-wave pulses; Not provided (individual); 200 μs; 20 Hz	TMS, STDT, tactile spatial acuity and short intracortical inhibition
Zarei et al. (41)	Case-matched study two group; pre-post test	40 healthy volunteers; Group A/(20n) TENS; Group B (20n) (Placebo)	TENS two blocks of 40 trials applied for 20 min	The electrical pulses were delivered through the same electrodes (4 × 4.6 cm) as used in the SEP procedure (left-MN of the non-dominant hand)	Constant current stimulator in the form of square-wave pulses below the motor threshold (individual); 1 ms; 100 Hz	EEG
**Studies made on stroke population**						
Bao et al. (12)	Retrospective case-matched study two groups; pre-post	Group A / BWSTT / 90 stroke patients; Group B / FES plus BWSTT/ 90 stroke patients	Group A / BWSTT for 30 min daily; Group B / FES for 45 min twice a day, plus BWSTT for 30 min daily for 8 weeks	FES of paretic leg 6 cm X 9 cm and 4 cm x 4 cm electrodes four output channels and a one-foot switch	Bidirectional symmetry square-wave pulses; 15 mA; 0.3 ms; 30 Hz	Walking speed, step length, step cadence, LL-fMa, CSS, 10MWt, TBT and MEP
Peurala et al. (13)	Case-matched study three-group; pre-post test	Group A / 32 stroke patients, active treatment of the paretic hand; Group B / 19 stroke patients, active treatment of the paretic foot; Group C/8 stroke patients, placebo treatment in the paretic hand	Group A and B active FES for 20 min twice a day Group C Placebo for 21 days	Cutaneous stimulation of paretic hand or paretic foot treatment 6 cm diameter electrode *via* glove/sock electrode	Monophasic constant current twin pulses; Not provided (individual); Not provided; 50 Hz	MMAS, 10MWt, paretic limb function, limb skin sensation
Giaquinto et al. (42)	Case-matched study two-group; pre-post test	Group A / 20 stroke patients; Group B / 82 stroke patients (control group)	Twice a day (morning and afternoon)	Target or non-target stimulation of the impaired or non-impaired hand, shoulder or hip using feedback system (electrode size is not provided)	Constant current pulses; 25 mA, above the threshold; 0.1 ms; Not provided;	CT scan and/or NMR, FIM, CIRS 14 and EEG signals
Tashiro et al. (15)	Case-matched study one-group; pre-post test	23 stroke patients	HANDS therapy system, applied for 8 h each day for 21 days	A hybrid electrode (10 mm diameter) for EMG detection and stimulation was placed on the belly of the affected EDC. An electrode (10 mm) for stimulation was placed on the affected EIP.	Not provided; Not provided (individual); Not provided (individual); Not provided (individual)	SWMT, TLT, FMA, MAS, SIAS, and MAL-14

BWSTT, Body Weight-Supported Treadmill Training; FES plus BWSTT, Functional Electrical Stimulation plus Body Weight-Supported Treadmill Training; FES, Functional Electrical Stimulation; tsDCS, Transcutaneous spinal (anodal and cathodal) direct current stimulation: MEP, Magnetic Evoked Potential; LL-fMa, Fugl-Meyer Lower-limb Scale; CSS, Composite Spasticity Scale; 10MWt, 10-Meter Walk Test; TBT, Tinetti Balance Test; MMAS, Modifed Motor Assessment Scale; NMR, Nuclear Magnetic Resonance; CT scan, Computed Tomography; FIM, Functional Independence Measure; CIRS 14, Cumulative Illness Rating Scale; EEG, Electroencephalography; HANDS, Hybrid Assistive Neuromuscular Dynamic Stimulation; EDC, Extensor Digitorum Communis; EIP, Extensor Indicis Propris; SWMT, Semmes–Weinstein Monofilament Test; TLT, Thumb Localizing Test; FMA, Fugl-Meyer Assessment for the UL; SIAS, Stroke Impairment Assessment Set; MAS, Modified Ashworth Scale; Mal-14, Motor Activity Log-14; TENS, Transcutaneous Electrical Nerve Stimulation; EA, Electroacupuncture; PES, Peripheral Electrical Stimulation; ABP, Abductor Pollicis Brevis; TMS, Transcranial Magnetic Stimulation; HF-RSS, High Frequency Repetitive Somatosensory Stimulation; STDT, Somatosensory Temporal Discrimination Threshold.

**Table 3:** Latencies and Amplitudes of SEP Components—Pre–Posttest—Studies made on healthy volunteers.

**Study/sample size**	**SEP components (latencies and amplitudes)**	**Test time/follow-up and statistical analysis**	**Significant effects**
Ashton et al. (6) 32 healthy volunteers	MN at the wrist, troughs and peaks utilizing latency criterion of: P1:60-100 msec, N1:100-160 msec, P2: 160-260 msec, N2 and P3: 260-360 msec	Pretreatment/- 15 min Post-I/0 min Post-II/+15 min Post-III/30 min Post-IV / +45 min 1-way ANOVA and 2-way ANOVA	Consideration of means showed a decrease of N1P2 amplitude and increase of N1 latency in the TENS group as compared to placebo or aspirin group. For the SEP total excursion measure, a significant effect occurred in the time epoch 30 min post-treatment (F = 3.92, df = 2, 29, *P <* 0.05) and a marginal effect in the last time epoch 45 min post-treatment (F = 2.79, df = 2, 29, 0.10 > *P >* 0.05).
Cogiamanian et al. (38) 12 healthy volunteers	The SEP of MN at the wrist: P14, N20 latency and amplitudes and TN SEPs at the ankle: N9, N22, P30, P39, latency and amplitudes	Baseline Post-I/0 min Post-II/+20 min 1-way ANOVA and 2-way ANOVA Post hoc analysis	Compering changes in TN and MN SEPs after anodal tsDCS over the thoracic spinal cord con- firmed that P30 component elicited by TN stimulation decreased by 49% in amplitude (baseline 0.78 ± 0.12 lV, T0 0.40 ± 0.07 lV; *t-* test: *p =* 0.01), but remained statistically unchanged in latency (baseline 28.8 ± 0.67 ms, T0 28.5 ± 0.57 ms; *t-*test: *p =* NS). After thoracic tsDCS all the median nerve SEP components remained unchanged (P14 amplitude: baseline 0.68 ± 0.10 lV, T0 0.70 ± 0.04 lV; *t-*test: *p =* NS; P14 latency: baseline 13.9 ± 0.48 ms, T0 13.7 ± 0.48 ms; *t-*test: *p =* NS).
Kang et al. (39) 20 healthy volunteers	The SEP of MN at the wrist: N13, N20, P25, N30 latency and amplitudes	Baseline During the stimulation period Post-I / + 20 min 1-way ANOVA and Scheffe's post hoc correction	EA demonstrated a higher mean amplitude in N20 during the stimulation and post- stimulation periods compared with baseline. In N30 the difference only appeared during the stimulation period when treated with EA. These effects were not observed when subjects were treated with sham TENS or 2 Hz TENS. No significant differences were observed in other components of MN-SEPs, either for mean latency or amplitude.
Schabrun et al. (33) 13 healthy volunteers	The SEP of MN at the wrist: peak-to-peak amplitudes: P14-N20, N20-P25, P25-N33, N13, N9 and latencies N9, N14 and N20	Before and after completion of the stimulation period 1-way ANOVA Linear regression analyses Where appropriate, *post-hoc* tests were performed	Neither motor or sensory PES induced a change in the latency of the N13/N20 1. Motor movement: Motor PES increased the amplitude N20-P25 (*post-hoc* pre vs. post *p =* 0.007,) no change in the P14-N20 (*post-hoc* pre vs. post *p =* 0.34) or P25-N33 (*post-hoc* pre vs. post *p =* 0.77) components. 2. Sensory 100 Hz: Sensory PES increased the amplitude of P14-N20 (*post-hoc* pre vs. post *p =* 0.01,) and reduced P25-N33 (*post-hoc* pre vs. post *p =* 0.001) The N20-P25 component was unchanged by sensory PES (*post-hoc* pre vs. post *p =* 0.34).
Rocchi et al. (40) 15 healthy volunteers	Digital nerves of the right index finger were stimulated The UL SEPs: amplitudes: P14, N20-P25 and N20 peak latency	Before and 5 min after the completion of the 45 min stimulation period 2-way ANOVA and dependent Student's *t-*test	HF-RSS increased the amplitude of N20-P25 (*p <* 0.001) and P14 (*p <* 0.001) immediately after HF-RSS was applied. No changes in N20 or P14 latency were observed (p values of all *t-* tests > 0.05)
Zarei et al. (41) 40 healthy volunteers	The SEP of MN at the hand: N100, P200, and N400 latency and amplitudes	Baseline Post-I / 0 min Post-II/+30 min Post-III/60 min 2-way ANOVA Where appropriate, *post-hoc* tests were performed	The magnitude of N100, P200 waves, and theta and alpha band power was significantly suppressed following the TENS intervention. The suppression of the magnitude of the N100 wave lasted at least an hour. However, the effects of TENS on the magnitude of P200 only remained for 30 min after the intervention.

MN, Median nerve; TENS, Transcutaneous Electrical Nerve Stimulation; EA, Electroacupuncture; UL, Upper limb; HF-RSS, High Frequency Repetitive Somatosensory Stimulation; LL, Lower limb; TN, Tibial nerve; tsDCS, Transcutaneous Spinal (anodal and cathodal) Direct Current Stimulatio.

**Table 4:** Latencies and Amplitudes of SEP Components—Pre–Posttest –Studies made on stroke population.

**Study/sample size**	**SEP components (latencies and amplitudes)**	**Test time/follow-up and statistical analysis**	**Significant effects**						
Bao et al. (12) 90 stroke patients	Not provided	Baseline, end of week 8 Paired *t-*tests and McNemar tests, 1-way ANOVA, χ2 tests	Significant differences in latency and peak value of SEP between the two groups at the end of the eighth week (*p <* 0.05), but not at baseline (*p >* 0.05).						
			**Latency (ms)**				**Peak (μV)**		
				**Baseline**	**8 weeks**	**P value**	**Baseline**	**8 weeks**	**P value**
			Group A	43.7 ± 5.56	38 ± 3.6	P < 0.05	1.44 ± 0.52	2.13 ± 0.51	P < 0.05
			Group B	44.1 ± 6.97	27.3 ± 5.36	P < 0.01	1.53 ± 0.46	2.94 ± 0.59	P < 0.01
			*P*-value	0.89	P < 0.01		0.7	P < 0.01	
Peurala et al. (13) 59 stroke patients	The SEP of MN at the wrist: N20, N30, N60, (patients with hand stimulation treatment) and TN SEPs at the ankle: P40, N80, (patients with foot stimulation treatment)	Baseline, end of week 3 Paired samples *t-*test, nonparametric Wilcoxon and marginal homogenity test	SEP normality classification improved significantly in paretic UL (*p <* 0.01) and in paretic LL (*p <* 0.05) in the stimulated group (n = 51) after 3 weeks of rehabilitation.						
			Hand SEP*(n **=** **8)**	**Before**	**After**	Foot SEP*(n **=** **19)**	**Before**	**After**	
			1	0	0	1	0	2	
			2	3	3	2	10	10	
			3	5	5	3	9	7	
			*SEP: 1, normal; 2, minor change; 3, abnormal						
Giaquinto, et al. (42) 102 stroke patients	The UL SEP N20 latency, affected and unaffected side	Baseline, end of week 8 Mann–Whitney *U*-test, Student's *t-*test, Spearman correlation	The mean amplitude N20 on the affected side increased compared to the baseline. Latencies did not change.						
			**N20: Mean Amplitude SD**			**N20: Mean Latencies and SD**			
				**Unaffected Hemisphere**	**Affected Hemisphere**	**Unaffected Hemisphere**		**Affected Hemisphere**	
			Before	−3.4 μV (1.5)	−1.8 μV (1.4) df = 18, *t =* 3.716, *P =* 0.002	20.5ms (1.5)		17.7ms (7.9) df = 18, *t =* 1.489, ns	
			After (1.3)	−3.4 μV	−2.6 μV (1.2) df = 16, *t =*−2.270, *P =* 0.003	20.1ms (1.2)		19.2ms (5.1) df = 16, *t =* 0.735, ns	
			Before and after comparison	df = 016, *t =* 0.363, ns df = 16, *t =* 4.932, *P =* 0.0001					
Tashiro et al. (15) 23 stroke patients	NI(N20), PI(P25), NII(N33), PII(P45), NIII(N60) of the MN at the wrist and N31, P35, N42, P53, N66 of the TN at the ankle	Baseline, end of week 3; Wilcoxon signed-rank tests Student's *t-*test	The Wilcoxon signed-rank test indicated that the number of cortical peaks significantly increased in the MN, but not in the TN in the non-paretic side (MN, *p =* 0.008; TN, *p =* 0.11), No significant changes in the MN between peak latencies of central SEP peaks and N18. Remarkable differences were detected in shortening of the latency between NI(N20)– PII(P45) after the intervention.						

MN, Median nerve; TENS, UL, Upper limb; HF-RSS, High Frequency Repetitive Somatosensory Stimulation; LL, Lower limb; TN, Tibial nerve.

### Exclusion/inclusion criteria

All type of non-randomized and randomized intervention studies were included. Moreover, intervention studies with no control group were also included since it was anticipated that the available data to answer the research question would be limited. No restrictions were set with regard to the body parts treated (UL, LL or torso) with PES. Studies in which only electroacupuncture was used were excluded since the piercing through dermis can affect additional neurological afferent pathways associated with pain giving misleading SEP results (43). Studies that measured SEP only during the intervention without follow-up measure were excluded since the study search is limited on SEP use as a change predictor. No limits were set regarding the outcome measures used to determine motor impairment and/or functional performance. Data from abstracts, letters, pilot studies, case studies and review articles were excluded from the study. Studies involving children and animals were not considered either. No limitations were applied regarding the type of stroke (ischemic or hemorrhagic), the time elapsed since the last occurrence, or the stroke location. In the text, the term stroke is used for both, ischemic and hemorrhagic stroke. The studies which focused on the effect of electrical stimulation on any of the following conditions were excluded: spinal cord injuries, Parkinson's disease, multiple sclerosis, pain or cranial nerve. Furthermore, the studies that used transcranial direct current stimulation, transcranial magnetic stimulation, or deep brain stimulation were excluded.

### Methodological quality

The Cochrane risk of bias in non-randomized studies (ROBINS-I) tool developed by Sterne et al. (44) was used to assess the risk of bias of observational studies that compare health effects of two or more interventions. ROBINS-I is a tool for evaluating risk of bias in estimates of the comparative effectiveness (harm or benefit) of interventions from studies that did not use randomization to allocate units (individuals or clusters of individuals) to comparison groups. ROBINS-I'S fundamental underlying principle is to compare the risk of bias associated with the current evaluated non-randomized trial with a target randomized controlled trial (RCT) hypothesized to be conducted with the same group of participants, even though this RCT may not be feasible or ethical (45). The ROBINS-I tool includes seven domains to assess the risk of bias that may arise in a non-randomized study: (1) bias due to confounding; (2) bias in selection of participants into the study; (3) bias in classification of interventions; (4) bias due to deviations from intended interventions; (5) bias due to missing data; (6) bias in measurement of outcomes (or detection bias); (7) bias in selections of the reported results. The categories for risk of bias judgments are Low risk, Moderate risk, Serious risk and Critical risk. The risk of bias is first assessed for each domain, and then the overall judgement of the study's risk of bias is made (44).

The “Quality Assessment Tool for Before-After (Pre-post)” developed by the National Institutes of Health (NIH) was used to rate the methodological quality of pre-post studies without a control group (46). The questions in the NIH quality assessment tool were designed to help reviewers focus on the key concepts for evaluating the internal validity of a study. Critical appraisal of a study involves considering the potential for selection bias, information bias, measurement bias, or confounding. Examples of confounding include co-interventions, differences at baseline in patient characteristics, and other issues addressed throughout the tool which can be found in Table 5. High risk of bias translates to a rating of poor quality; low risk of bias translates to a rating of fair and good quality (46).

**Table 5:** Methodological quality of included studies according to the “Quality Assessment Tool for Before-After (Pre-post)”.

	**Studies made on healthy volunteers**				**Study made on stroke population**
	**Cogiamanian et al. (38)**	**Schabrun et al. (33)**	**Kang et al. (39)**	**Rocchi et al. (40)**	**Tashiro et al. (15)**
1. Was the study question or objective clearly stated?	Yes	Yes	Yes	Yes	Yes
2. Were eligibility/selection criteria for the study population prespecified and clearly described?	Yes	Yes	Yes	Yes	Yes
3. Were the participants in the study representative of those who would be eligible for the test/service/intervention in the	Yes	Yes	Yes	Yes	Yes
general or clinical population of interest?					
4. Were all eligible participants that met the prespecified entry criteria enrolled?	Yes	Yes	Yes	Yes	Yes
5. Was the sample size sufficiently large to provide confidence in the findings?	No	No	No	No	Yes
6. Was the test/service/intervention clearly described and delivered consistently across the study population?	Yes	Yes	Yes	Yes	Yes
7. Were the outcome measures prespecified, clearly defined, valid, reliable, and assessed consistently across all study	Yes	Yes	No	Yes	Yes
participants?					
8. Were the people assessing the outcomes blinded to the participants' exposures/interventions?	Yes	Not reported	Not reported	Not reported	Yes
9. Was the loss to follow-up after baseline 20% or less? Were those lost to follow-up accounted for in the analysis?	Yes	Not reported	Yes	Yes	Yes
10. Did the statistical methods examine changes in outcome measures from before to after the intervention? Were statistical tests were done that provided *p*-values for the	Yes	Yes	Yes	Yes	Yes
pre-to-post changes?					
11. Were outcome measures of interest taken multiple times before the intervention and multiple times after the intervention (i.e., did they use an interrupted time-series	Yes	No	Yes	Yes	No
design)?					
12. If the intervention was conducted at a group level (e.g., a whole hospital, a community, etc.) did the statistical analysis take into account the use of individual-level data to	No	NA	NA	NA	NA
determine effects at the group level?					
Quality rating	Fair	Fair	Poor	Fair	Good

The overall certainty of evidence and strength of recommendation was assessed using the Grades of Recommendation Assessment, Development and Evaluation (GRADE handbook) methodology (47). According to the GRADE approach, the evidence is graded as high, moderate, low, or very low certainty of evidence. Furthermore, a body of evidence from observational studies begins with a low certainty of evidence-rating which could be downgraded due to five reasons: risk of bias, indirectness, inconsistency, imprecision and publication bias (36). There are three factors that permit rating up the certainty of evidence: large magnitude of an effect, dose-response gradient, and effect of plausible residual confounding (47).

## Results

**Figure 1:**
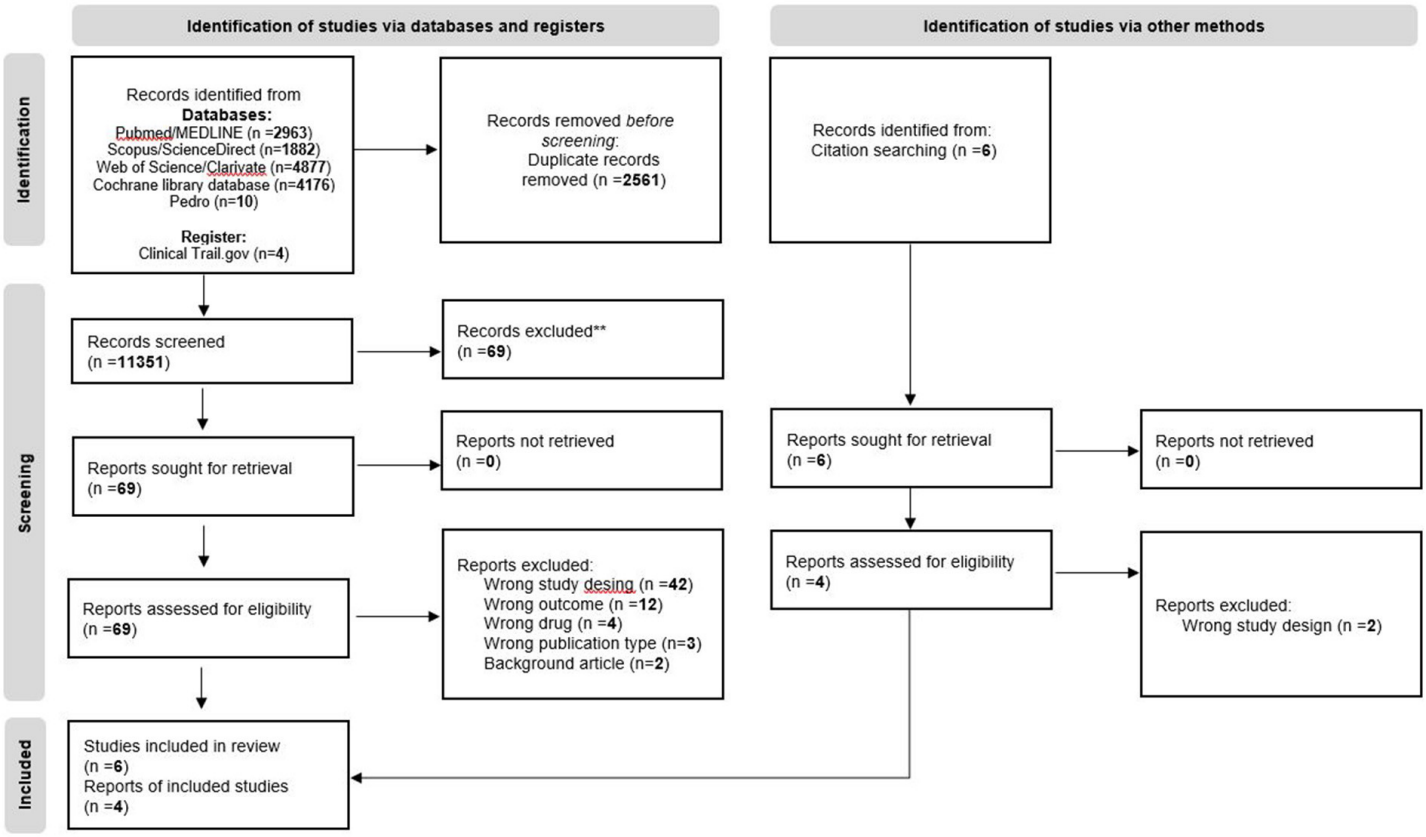
PRISMA flow chart.

The systematic search resulted in 11,351 references. The search from Pubmed/MEDLINE database resulted in 2,963 records, Scopus/ScienceDirect database resulted in 1,882 records, Cochrane library database resulted in 4,176 records, Web of Science/Clarivate resulted in 4,877 records and the database PEDro resulted in 10 records. The registry ClinicalTrials.gov was searched manually, and four studies were included for further evaluation. We excluded 2,561 duplicate studies using Rayyan QCRI software (37). Based on the titles and abstracts 69 reports were included for full-text reading. Additionally, six articles were found after the screening of reference lists. Ten articles were included in the review after applying the inclusion and exclusion criteria. The search process is presented in the PRISMA flow diagram (Figure 1).

### Intervention procedure and the time frame between the SEP measurements

The different forms of stimulation, stimulation devices, location of PES, amplitude, duration, and frequency pulse as well as test time and follow-up of SEP were examined in each study. All data are summarized in Table 2. The total amount of participants in the reviewed studies was 496. Of these, 364 were stroke patients and 132 were healthy participants. Five studies involved one (12, 41, 42) or two (6, 13) control groups. The study by Kang et al. (39) applied: Sham TENS, 2Hz TENS or 2Hz electroacupuncture and the study from Schabrun et al. (33) applied Motor Movement PES or Sensory PES 100 Hz intervention. On the other hand Tashiro et al. (15) used SEP of the tibial nerve as a reference to SEP for the median nerve and Cogiamanian et al. (38) measured five subjects from the first group a second time using sham stimulation. A wide range of sensory-motor assessments was used to examine the effect of PES in studies of stroke patients and healthy participants (Table 2). The assessment of SEP in two studies on stroke patients (12, 42) was performed at baseline and 8 weeks post PES intervention. In the other two studies (13, 15) the assessment was performed at baseline and 3 weeks post PES intervention. The SEP in healthy participants in all six studies was assessed before, at baseline, and 0,15/20/30/45/60 min after the intervention (6, 33, 38–41). In the majority of studies on stroke patients the SEP measurements were performed on the median nerve. In the study by Peurala et al. (13) SEPs on the UL were performed in those patients who received hand stimulation and SEPs on the LL were performed in those patients who received foot stimulation while in the study from Tashiro et al. (15) SEPs from tibial nerves were used as a control measurement. Bao et al. (12) reported an improvement of latency and peak value of SEPs between the two groups at the end of the 8th week without further explanation of how the measurement was performed. No study showed a loss of peaks after the intervention. Details about SEP changes in latencies and amplitudes components can be found in Tables 3, 4. The following body location were stimulated with PES: tenth thoracic spinal vertebra (38), shoulder (38, 42), arm, hand or fingers (6, 13, 15, 33, 40–42), lower limb and foot (12, 13, 39) and hip (42). The found data about stimulation form, stimulation devices, location of PES, amplitude, duration, and frequency pulse as well as test time and follow-up of SEP could not be standardized so we decided to analyzed data separately.

### Methodological quality of included studies

**Figure 2:**
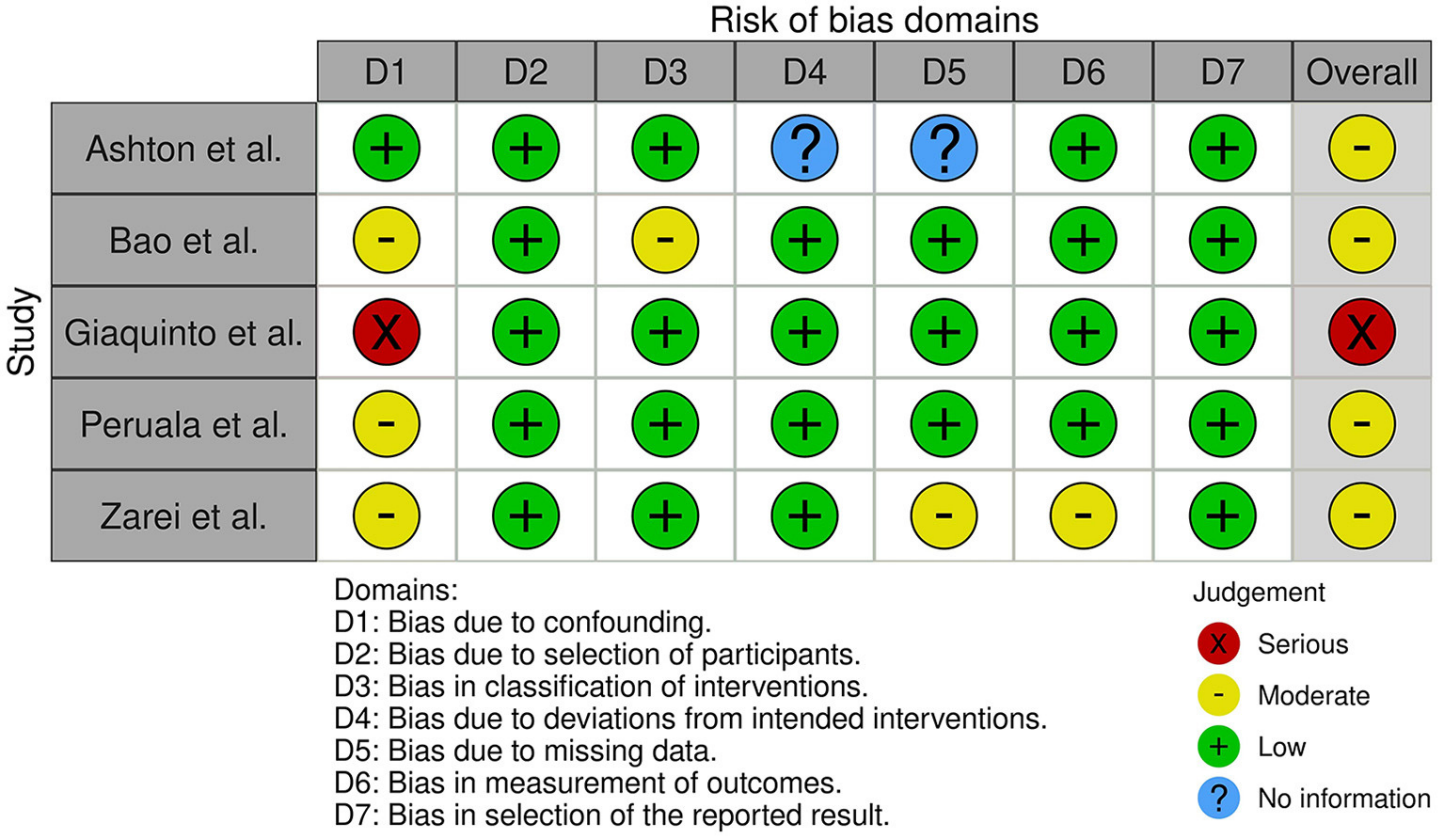
Results of the ROBINS-I tool to assess risk of bias domains.

Five non-randomized studies (6, 12, 13, 41, 42) with serious to moderate risk of bias (Figure 2) and five pre-post studies (15, 33, 38–40) without a control group with poor to good methodological quality (Table 5) were included and assessed in the present review. No randomized trials were found. Low overall risk of bias in non-randomized trials, which can be compared to a well-performed randomized trial, was not found in any of the included studies (6, 12, 13, 41, 42). Due to the fact that the Robins-I tool uses strict criteria to evaluate confounding bias, one study was classified as having serious risk of bias (42). Four studies were evaluated as having moderate risk of bias because the data was collected retrospectively (12), insufficient information was given about the potential confounding bias (13, 41), or no explanation of the source of information about intervention status was reported (6). In accordance with the NIH tool, one pre-post study had good (15), tree studies had fair (33, 38, 40), and one had poor (39) methodological quality. The eligibility criteria and the outcome measures were prespecified, clearly defined, valid, reliable, and assessed consistently across all study participants, except for the study by Kang et al. (39). According to the NIH tool, the sample size should be large enough to provide confidence in the findings and outcome assessors should be blinded to the participants' exposures, and interventions. Only the study by Tashiro et al. (15) managed to meet these important criteria.

The overall certainty of evidence was very low for all outcomes (Supplementary Table 1). All available evidence was downgraded for limitations in study design and risk of bias (6, 12, 13, 15, 33, 38–42), imprecision (6, 12, 13, 15, 33, 38–42), or indirectness (6, 12, 39, 41) to very low certainty of evidence. There were no legit reasons to rate up the certainty of evidence. Thus, there is insufficient evidence for or against the use of SEP to monitor therapeutic effects.

### Synthesis of results

It was planned to perform a meta-analysis, if enough homogenous data were available, using mean difference and random effects model. However, quantitative synthesis was not possible due to limited and heterogenous eligible studies. Therefore, a qualitative synthesis was performed. Table 6 features a summary of SEP latency and amplitude outcomes in studies using same measurement instruments.

**Table 6:** SEP latency and amplitude outcomes synthesis in studies using same measurement instruments.

**Outcomes/study**	**Measurement instruments**	**Significant effects**
**Studies made on stroke population**		
**Amp N20/P25/UL** (13, 15, 42)	The UL SEP N20 amplitude was recorded using surface electrodes placed in anatomically identified locations of the hand area of the primary somatosensory cortex. Affected and unaffected side was measured.	The signal amplitude N20 **increased**. Student's *t-*test (*P =* 0.0001) (42) SEP normality classification **improved** significantly in paretic UL. Paired samples *t-*test (*p <* 0.01) (13) The number of cortical peaks **increased** significantly The Wilcoxon signed-rank test (*p =* 0.008) (15)
**Lat N20/UL** (13, 15, 42)	The UL SEP N20 latency was recorded using surface electrodes placed in anatomically identified locations of the hand area of the primary somatosensory cortex. Affected and unaffected side was measured.	Latencies **did not change**. Student's *t-*test (*p >* 0.05) (42) SEP normality classification **improved** significantly in paretic UL. Paired samples *t-*test (*p <* 0.01) (13) **No significant changes** between peak latencies (*p >* 0.05) (15)
**Amp P40/LL** (12, 13)	Not provided (12) The TN SEP P40 amplitude was recorded using surface electrodes placed in anatomically identified locations of the LL area of the primary somatosensory cortex. Affected and unaffected side was measured (13)	The signal amplitude N20 **increased**. Paired samples *t-*test (*p <* 0.05) (12) SEP normality classification **improved** significantly in paretic LL. Paired samples *t-*test (*p <* 0.05) (13)
**Lat P40/LL** (12, 13)	Not provided (12) The TN SEP P40 latency was recorded using surface electrodes placed in anatomically identified locations of the LL area of the primary somatosensory cortex. Affected and unaffected side was measured (13)	Latency **improved** after intervention. Paired *t-*tests (*p <* 0.05) (12) SEP normality classification **improved** significantly in paretic LL. Paired samples *t-*test (*p <* 0.05) (13)
**Studies made on stroke population**		
**Amp N20/P25/UL** (33, 38–40)	The UL SEP and N20/P25 amplitude was recorded using surface electrodes placed in anatomically identified locations of the hand area of the primary somatosensory cortex.	The signal amplitude **decreased**. paired *t-*tests *p =* 0.01 (38) **No significant differences** observed in amplitude. One-way analysis of variance shown as mean±SD (39) Motor PES **increased** the amplitude (*post-hoc* pre vs. post *p =* 0.007,) (33) The signal amplitude **increased**. Dependent *t-*tests were (*p <* 0.001) (40)
**Amp N100/UL** (6, 41)	The EEG signals was recorded during the sensory evoked potential (SEP) phases.	The signal amplitude **decreased**. 2-way ANOVA (*P >* 0.05) (6) The magnitude was significantly **decreased**. 2-way ANOVA (*P >* 0.05) (41)
**Lat N100/UL** (6, 41)	The EEG signals was recorded during the sensory evoked potential (SEP) phases.	Latency **increased** after intervention. 2-way ANOVA (*P >* 0.05) (6) Latencies **did not change**. 2-way ANOVA (*P >* 0.05) (41)

UL, Upper limb; LL, Lower limb; Amp, Amplitude: Lat, Latency.

## Discussion

### Studies made on healthy volunteers

By evaluating motor function and control, corresponding to changes in SEP components in healthy participants, the study by Rocchi et al. (40) suggested that high frequency repetitive somatosensory stimulation leads to improved performance in behavioral tests of temporal discrimination and contributes to improved performance in tests of spatial detection. Nevertheless, high frequency repetitive somatosensory stimulation also affects short-latency inhibition in M1. Together these changes in S1 and M1 may underlie reported improvements in manual motor performance (40). The correlation in healthy individuals between SEP and similar neurophysiological procedures as described in the study by Schabrun et al. (33). The magnitude and direction of the change in corticomotor excitability induced by sensory and motor PES was positively correlated with the difference in the cortical SEP components (*r* = 0.71, *p* < 0.001), as confirmed by linear regression between cortical SEP components (N20-P25 and P25-N33) and corticomotor excitability motor evoked potential (MEP) amplitude. Similar changes as already described in the study from Rocchi et al. (40) showed a correlation between PES, high-frequency oscillations analysis and N20-P25 recovery curve. The first conclusion considered from the obtained results is a good validity between SEP, TMS and its correlation with PES. In other terms, not only somatosensory brain areas are affected through PES. Moreover, the motoric brain regions are part of this process. Despite those fact it is necessary to note that all studies made on healthy volunteers assess SEP maximum 1 h after PES and that the studies (38, 41) showed selectively reduced amplitudes in primary somatosensory cortex direct after stimulation. However, we did not identify enough data to provide a clear relationship between SEP and motor performance subsequently to PES in healthy individuals. The lack of observation studies over extended periods of time is the main problem when it comes to drawing a clear conclusion about the impact of PES on SEP.

### Studies made on stroke patients

In the stroke study (13) SEP and Modified Motor Assessment Scale results were not compared, but both measures showed improvement. Moreover, in a study (42) significant negative correlation between the time interval for the appearance of somatosensory event-related potentials and the functional independence measure (FIM) score at the time of discharge (*r* = −0.53, *p* < 0.01). The study on stroke patients by Tashiro et al. (15) observed significant improvements in behavioral assessment scores for proprioception followed by PES interventions. We could conclude that assessments of motor performance correspond to changes in SEP components in UL and LL. Additionally, the studies on stroke patients involving PES that initiate a voluntary contraction used for a specific movement or task (12, 13, 15, 42), indicate a positive relationship and correlation to assessments of motor function. This hypothesis is supported by findings in a meta-analysis on stroke motor recovery of UL functions (48) and therapeutic effects of peroneal stimulation on gait and motor recovery (18). Moreover, simple sensory stimulation, unrelated to the movement, was of limited functional value for motor recovery for the rehabilitation of the hand in stroke patients and no correlation was described (49).

### SEP results in healthy subjects compared to stroke patients

Somatosensory event-related potentials accompanied with SEP in a study from Giaquinto et al. (42) were adequate to follow changes in primary somatosensory area N20. The Bao et al. (12) found significantly improved latency and peak value of SEP and MEP. Furthermore, those changes respond to sensory and motor nerve conduction velocity at the end of the 8 week (*p* < 0.05). This finding indicates the relevance of evaluating electrophysiological methods and may verifies the use of SEP in stroke patients. All SEP set on stroke patients demonstrated several subcortical or cortical reorganization changes after treatment with PES on the paretic side. However, an unrelated time frame and insufficient data were collected to analyze the relationship between the form of stimulation, pulse amplitude, pulse duration, or location of stimulation and changes in SEP. Perhaps it should be emphasized that in stroke studies in which high pulse amplitude inducing muscle contraction was delivered, the increase of amplitude N20-P25 (15, 42) was seen. The same was observed in healthy participants (33, 40). In order to confirm this hypothesis, we suggest conduction of randomized controlled study on healthy subjects and stroke patients using standard SEP procedure define by Muzyka et al. (31), and clearly described used PES parameter. Based on the GRADE approach to assess the quality of evidence, no outcome that provided a strong recommendation was found. There is thus far insufficient evidence to support a decision for or against use of SEP to monitor therapeutic effects and the results of this analysis cannot be generalizable.

It is known that there are substantial anatomical interconnections linking the brain's motor and somatosensory regions. Cortical motor areas receive direct inputs from primary and second somatosensory cortex and inversely, somatosensory areas get direct cortical inputs from primary motor cortex, premotor cortex, and from supplementary motor area (21, 50). A change in somatosensory function in association with motor learning would seem to be a natural by-product of this anatomical connectivity (21, 50). Findings in this review suggest that PES may shift the response of somatosensory to motor areas of the brain. On the other hand, it could be hypothesized that SEP can indirectly recognize the changes in motor area of the brain. Moreover, it appears that SEPs have sufficient sensitivity to detect even the smallest changes in action potential of neural cortical network after stroke and is probably able to assess the effect of various sensory therapies: cryotherapy, thermotherapy, occupational tactile therapy, or robotic tactile therapy more directly.

## Limitations of the study

First, we cannot confirm that all PES studies were identified because the meaning of the term “peripheral electrical stimulation” varies widely and is understood differently. We tried to minimize this limitation by searching more databases. Second, we were aware that EEG measurement can also be used to record SEP, and the lack of keywords and terms to describe this process limited our desire to include all of these studies. However, we used the term “evoked potentials” to increase the number of studies identified in our database search and to include studies using EEG. We also tried to use all PES terms indexed in PubMed to find an optimal data set and minimize this limitation.

## Conclusion

From the results of this review, the repetitive task-oriented treatment enriched with PES could likely become a different approach to be applied in stroke patients to improve daily living activities since we have hints that PES may impact changes in motor neuroplasticity. We suggest that more studies (especially RCTs) should be conducted to evaluate whether SEP measures can be used to monitor the therapeutic effects of PES in the rehabilitation of stroke patients, as there is insufficient evidence to do so but SEP remains a promising tool to estimate rehabilitation prognosis after stroke.

## Data availability statement

The original contributions presented in the study are included in the article/Supplementary material, further inquiries can be directed to the corresponding author.

## Author contributions

MM, AJ, BS, and PY contributed to conception and design of the study. MM organized the database and wrote the first draft of the manuscript. MM and AJ performed the qualitative analysis. All authors contributed to manuscript revision, read, and approved the submitted version.

## Conflict of interest

The authors declare that the research was conducted in the absence of any commercial or financial relationships that could be construed as a potential conflict of interest.

## Publisher's note

All claims expressed in this article are solely those of the authors and do not necessarily represent those of their affiliated organizations, or those of the publisher, the editors and the reviewers. Any product that may be evaluated in this article, or claim that may be made by its manufacturer, is not guaranteed or endorsed by the publisher.

## Abbreviations

PES: peripheral electrical stimulation
TENS: transcutaneous electric nerve stimulation
FES: functional electrical stimulation
SEP: somatosensory evoked potentials
UL: upper limb
LL: lower limb
CNS: central nerve system
MEP: motor evoked potential
FIM: Functional Independence Measure
NIH: National Institutes of Health
RCT: randomized controlled trial.
