# Supplementary material for: Use of peripheral electrical stimulation on healthy individual and patients after stroke and its effects on the somatosensory evoked potentials. A systematic review
Source: Front Neurol. 2022 Nov 18;13:1036891. doi: 10.3389/fneur.2022.1036891 (PMC9716063; doi:10.3389/fneur.2022.1036891)
Supplement: Supplementary file 1 [file Table_1.pdf]

Supplementary Table 1. Certainty of evidence according to GRADE: Influence of PES on SEP latency and amplitude of median nerve and tibial nerve

Patients or population: Stroke patients or healthy volunteers
Settings: Somatosensory evoked potentials
Intervention: Peripheral electrical stimulation
Comparison: No PES intervention, placebo, or control group

Outcomes	Number of participants (studies)	Quality of evidence (Grade)
Stroke patients<br>**Amp N20/P25/UL** (1–3)	Case-matched studies:<br>N=20 (1)<br>N=23 (2)<br>N=29 (3)	Very low ⊕◯◯◯<br>Study design and risk of bias (1–3)<br>Indirectness of evidence (3)
Stroke patients<br>**Lat N20/UL** (1–3)	Case-matched studies:<br>N=20 (1)<br>N=23 (2)<br>N=29 (3)	Very low ⊕◯◯◯<br>Study design and risk of bias (1–3)<br>Indirectness of evidence (3)
Healthy volunteers<br>**Amp N20/P25/UL** (4–7)	One-group; pre-post test<br>N=12 (4)<br>N=13(6)<br>N=20 (5)<br>N=15 (7)	Very low ⊕◯◯◯<br>Study design and risk of bias (4–7)<br>Imprecision (4–7)<br>Indirectness of evidence (5)
Stroke patients<br>**Amp P40/LL** (3,8)	Case-matched study<br>N=29 (3)<br>N=90 (8)	Very low ⊕◯◯◯<br>Study design, risk of bias and indirectness of evidence (3,8)
Stroke patients<br>**Lat P40/LL** (3,8)	Case-matched study<br>N=29 (3)<br>N=90 (8)	Very low ⊕◯◯◯<br>Study design, risk of bias and indirectness of evidence (3,8)
Healthy volunteers<br>**Amp N100/UL**<br>(9,10)	Case-matched study<br>three group; pre-post test<br>N=32 (9)<br>Case-matched study<br>two group; pre-post test<br>N=40 (10)	Very low ⊕◯◯◯<br>Study design and risk of bias, imprecision (9,10)
Healthy volunteers<br>**Lat N100/UL** (9,10)	Case-matched study<br>three group; pre-post test<br>N=32 (9)<br>Case-matched study<br>two group; pre-post test<br>N=40 (10)	Very low ⊕◯◯◯<br>Study design and risk of bias, imprecision (9,10)

Abbreviations: UL, Upper limb; LL, Lower limb; Amp, Amplitude: Lat, Latency

Reference:

1. Giaquinto S, Mascio M, Fraioli L. The physiopathological bases of recovery processes: The bases of stroke rehabilitation. The cassino project. Clin Exp Hypertens. 2002;24(7–8):543–53.

2. Tashiro S, Mizuno K, Kawakami M, Takahashi O, Nakamura T, Suda M, et al. Neuromuscular electrical stimulation-enhanced rehabilitation is associated with not only motor but also somatosensory cortical plasticity in chronic stroke patients: an interventional study. Ther Adv Chronic Dis. 2019;10.

3. Peurala SH, Pitkänen K, Sivenius J, Tarkka IM. Cutaneous electrical stimulation may enhance sensorimotor recovery in chronic stroke. Clin Rehabil. 2002;16(7):709–16.

4. Cogiamanian F, Vergari M, Pulecchi F, Marceglia S, Priori A. Effect of spinal transcutaneous direct current stimulation on somatosensory evoked potentials in humans. Clin Neurophysiol [Internet]. 2008;119(11):2636–40. Available from: http://dx.doi.org/10.1016/j.clinph.2008.07.249

5. Kang YT, Liao YS, Hsieh CL. Different effects of transcutaneous electric nerve stimulation and electroacupuncture at ST36-ST37 on the cerebral cortex. Acupunct Med. 2015;33(1):36–41.

6. Schabrun SM, Ridding MC, Galea MP, Hodges PW, Chipchase LS. Primary Sensory and Motor Cortex Excitability Are Co-Modulated in Response to Peripheral Electrical Nerve Stimulation. PLoS One. 2012;7(12):1–7.

7. Rocchi L, Erro R, Antelmi E, Berardelli A, Tinazzi M, Liguori R, et al. High frequency somatosensory stimulation increases sensori-motor inhibition and leads to perceptual improvement in healthy subjects. Clin Neurophysiol [Internet]. 2017;128(6):1015–25. Available from: http://dx.doi.org/10.1016/j.clinph.2017.03.046

8. Bao X, Luo JN, Shao YC, Tang ZQ, Liu HY, Liu H, et al. Effect of functional electrical stimulation plus body weight-supported treadmill training for gait rehabilitation in patients with poststroke: A retrospective case-matched study. Eur J Phys Rehabil Med. 2020;56(1):34–40.

9. Ashton H, Golding JF, Marsh VR, Thompson JW. Effects of transcutaneous electrical nerve stimulation and aspirin on late somatosensory evoked potentials in normal subjects. Pain. 1984;18(4):377–86.

10. Zarei AA, Jadidi AF, Lontis ER, Jensen W. Short-term suppression of somatosensory evoked potentials and perceived sensations in healthy subjects following tens. IEEE Trans Biomed Eng. 2021;68(7):2261–9.
